# After-shift Musculoskeletal Disorder Symptoms in Female Workers and Work-related Factors: A Cross-sectional Study in a Seafood Processing Factory in Vietnam

**DOI:** 10.3934/publichealth.2016.4.733

**Published:** 2016-09-14

**Authors:** Thuy Thi Thu Tran, Chinh Thi Thuy Phan, Tuan Cong Pham, Quynh Thuy Nguyen

**Affiliations:** 1 Department of Occupational Health and Safety, Hanoi School of Public Health, Hanoi, Vietnam; 2 National Institute of Occupational and Environmental Health, Vietnam; 3 Centre for Environment and Population Health, Griffith University, Australia

**Keywords:** musculoskeletal disorder symptoms, female/women workers, seafood processing sector/factory, Vietnam, work-related factors, task demands, work organization, cold and humid working environment, vibration, neck, hips, back, extremities/limbs, shoulders

## Abstract

**Background:**

The seafood processing industry has been developing and providing marked contribution to Vietnam's economic growth in recent years. However, information on working conditions and their impacts to workers' health in this sector, focusing on musculoskeletal problems in female workers, has been poorly documented.

**Objectives:**

This paper examines the prevalence of after-shift musculoskeletal disorder symptoms (A-MSDS) and work-related factors in female workers in a seafood processing factory in Vietnam.

**Materials and Methods:**

As part of a comprehensive study, a cross-sectional survey was implemented in one seafood processing factory in the center of Vietnam in 2014. A self-administered questionnaire was completed by 394 female workers to collect information about their A-MSDS state, demographic characteristics, health status and work conditions. Descriptive analysis and logistic regression were applied to describe and analyse the results.

**Results:**

Nearly four-fifths of female workers experienced MSDs in at least one body part (77.7%) and 20.1% of them had MSDs in all investigated regions. The prevalence of A-MSDS in different body parts markedly varied, with the proportion of pain in the hips and lower extremities being as high as 53.3%, followed by pain in the shoulders and upper extremities (42.6%) and the neck (41.1%). A humid working environment, exposure to vibration and chemicals as well as taxing task demands and work organizations were found to significantly contribute to the increased risk of after-shift musculoskeletal disorders in female workers.

**Conclusion:**

Approximately 80% of female workers in the seafood processing factory experienced musculoskeletal pains after work, especially in the hips, extremities, neck and shoulders which were contributed by work conditions and task demands.

## 1. Introduction

Work-related musculoskeletal disorders (MSD) are one of the most common causes of health complaints in various sectors [1],[2]. Work-related MSDs account for significant costs of sick leave, loss of productivity, disability and increases in health care costs and labour compensation [1]. It is estimated by the Institute of Medicine that the annual economic burden of work-related MSDs ranges from $45 to $54 billion in the United States [3]. A similar issue was reported in the United Kingdom. According to the Health and Safety Executive, in 2014 and 2015, the days away from work due to work-related MSDs accounted for 40% of the total days away from work because of all work-related illnesses and injuries [4]. In addition, work-related MSDs are known to cause a variety of adverse effects on workers' health, such as chronic pain, reduced cognitive performance leading to loss of concentration [5], limitations to functional ability which make the most basic daily activities difficult [6] or even reduction in workers' quality of life [4].

In epidemiological studies, the term “musculoskeletal disorders” referred to a wide range of inflammatory and degenerative conditions affecting the muscles, tendons, ligaments, joints, peripheral nerves, and supporting blood vessels [5]. The underlying causes of discomfort or pain and disability may include damages of soft tissue structures and/or the joints or bones and/or associated connective tissues [6]. A combination of occupational hazards and personal variables for MSDs/work-related MSDs has been documented, for example, exposure to movement (frequency, velocity, acceleration, duration), force (frequency, magnitude), awkward angles and postures of body parts and vibration [5],[6], which can be observed in a diversity of occupations. The seafood processing sector is one of few industries in which high rates of work-related MSDs in different body parts have been widely reported, especially in female workers [7]–[10]. Studies in this sector also published a significant relationship between MSD prevalence and several typical working conditions, namely working in a cold environment [11],[12], performing repetitive and monotonous tasks [13],[14] and frequently lifting or carrying heavy objects [8],[9].

With the abundance of water resources and the diversity of fresh water and marine species, the fishery industry in general and the seafood processing sector in particular have made a significant contribution to Vietnam's economic growth in recent decades. The export value of seafood products has become the 5th largest source of Vietnam's GDP after electronics, garments, crude oil and footwear [15]. The sector also provides significant employment opportunities, especially for a female manual labour force in rural areas [15]. Additionally, a remarkable rate of increase in the number of seafood processing companies with modern production lines and heavy freezers to preserve product quality has been reported [16]. However, insufficient information on workers' health impacts in general and female workers' health conditions in particular, especially work-related MSDs issues, is available in Vietnam. Hence, the objective of this paper is to report one set of findings from a comprehensive study on the health status of female workers in industrial zones in Vietnam, focusing on the prevalence of after-shift musculoskeletal disorder symptoms (A-MSDS) in female workers in a seafood processing factory and work-related factors.

## 2. Materials and Methods

### 2.1. The Original Comprehensive Study

#### 2.1.1. Sampling and Data Collection

With the aim to describe the health status of female workers in industrial zones in Vietnam, the original study was conducted in ten factories in three industrial zones in three cities/provinces that represented the three main economic regions of this country where factories of the four light industrial sectors with the highest proportion of female workers (namely garment, footwear, seafood processing and electrical device manufacturing industries) were located [17],[18]. Therefore, the original survey selected four factories (all industries) in Hung Yen province (Northern region), three factories (garment, electrical device and seafood processing) in Da Nang city (Central region) and three factories (garment, electrical device and footwear) in Dong Nai province (Southern region). These cities/provinces were also selected for their increasing industrial development and a high in-migration rates [19].

The sample size for each province was calculated with the formula to estimate a proportion with absolute precision. The following parameters were considered for calculating the sample size: an anticipated prevalence of health problems among female workers as 35% [20], an absolute precision of 0.5%, an estimated non-respondent rate of 10% and a design effect of 2. Hence, the estimated number of female workers in each province was 950, in other words, a minimum of three factories with 320 female workers each would be invited to participate into the survey.

In each of the factories, the study's subjects were randomly selected following a systematic procedure from the list of female workers who had an official labour contract and had worked in the production lines of these factories for at least three months prior to the survey. In total, 2,818 female workers participated into this study, 940 from Hung Yen, 955 from Da Nang and 923 from Dong Nai.

The quantitative data was collected via a set of self-administrated instruments developed specifically for the purposes of this study, covering questions on the workers' social and demographic characteristics, income and living conditions, housing status, working conditions and health status. Additional information was gathered via in-depth interviews and group discussions with authorities, health and safety staffs and female workers. All quantitative questionnaires and qualitative instructions were pre-tested and revised according to the study's objectives.

#### 2.1.2. Data Management and Analysis

The analysis of quantitative data included descriptive statistical analysis (such as frequencies, percentage and cross-tabulation) and Chi-Square tests to identify potential relationships. All the analysis was carried out using SPSS software version 16 and at a 95% confidence interval. Qualitative data was described in themes to provide further explanation for quantitative findings.

#### 2.1.3. Ethics Approvals

This study obtained ethics clearance approval from the Institutional Review Boards of Hanoi School of Public Health (IRB reference number 171/2013/YTCC-HD3). Participation in the study was completely voluntary. Printed standard consent forms for participation were obtained from all eligible respondents at the beginning of the survey.

### 2.2. The Contents of this Paper

#### 2.2.1. Sampling and Data Collection

This paper exacted quantitative data of one seafood processing factory in Da Nang city, in the center of Vietnam, from the original study's database which was described above. A total of 394 female workers' profiles were suitable for data analysis.

From these profiles, outcome/dependent variables on musculoskeletal disorder symptoms after work in five body's regions, namely the neck, shoulders and upper extremities, upper back, lower back and hips and lower extremities, were collected. The presence of pain in each body part was recorded based on yes-no questions. A severity of pain/discomfort in specific parts was categorized in three levels: light, mid and severe.

In this article, several working conditions which were found to be significantly related to MSDs/work-related MSDs in the literature review were selected as independent work-related factors. The exposure to potential work-related factors such as coldness or vibration at work was recorded based on yes-no questions. Additionally, other personal characteristics with potential relationship with MSDs/work-related MSDs were also included in the analysis to control confounders such as marital status, number of children, years of service and history of injury.

#### 2.2.2. Data Management and Analysis

Descriptive statistical parameters were used such as frequency, percentage and mean to provide a further description of this paper's sample and prevalence of A-MSDS.

To analyse the relationship between the presence of A-MSDS and work-related risk factors, the additional logistic regression was performed to calculate adjusted OR.

#### 2.2.3. Job Description

Field observation and the factory's profile showed that manual work played the main role in the work of seafood processing. The main product of the factory was shrimp, while fish and squid were less common because of seasonal availability and business contracts. Therefore, workers were mainly exposed to the hazards of shrimp processing.

In order to export products to other countries, the production had to follow strict procedures to ensure that international standards on food quality and safety were met. Hence, shrimp was preserved in cold conditions from the start to the end of the processing line with minced ice. They were rinsed under cold water of 6 °C several times with or without chemicals (mainly chlorine). The daily indoor temperature varied from 12 °C to 18 °C during most of the working hours. Additionally, workers had to keep a constantly high pace so that the final products could be quickly stored in the freezer, because shorter processing time would better maintain the shrimp's quality.

The job primarily required the movements of workers' upper body and back in carrying and rinsing baskets and moving trays of shrimp. The shrimp containers' usual weights varied from two kilograms (in the weighing step) to ten kilograms (in the size-classification step). The use of rubber gloves required workers to apply more force in holding tools (e.g. a small knife) to perform accurate tasks on such small and slippery objects without damaging the meat (e.g. removing the carapace or whole head, removing part or whole body shells). Workers remained standing most of the work shift on unadjusted platforms and tables regardless of their height. Occasionally workers had to bend over to lift up or put down containers of products.

The factory adapted the productivity based wage regime; therefore, apart from the one-hour lunch break in the middle of the shift, only few short breaks for either resting or using toilet was observed among female workers. The average daily shift lasted 8 hours. However, it would increase to the peak of 10 to 12 hours per shift when required.

## 3. Results

### 3.1. Characteristics of the Study Participants

Table 1 presents the distribution of participants by several socio-demographic characteristics. The mean age of female workers was approximately 39 years old (SD ± 8, range 18–63). Nearly 5% of women were older than the national legal working age (>50 years old for female workers). Two thirds of the women finished high school (72.6%). Nearly 90% of female workers were in a marital relationship and over 83% of the study sample already had children. A quarter of female workers reported that they had to work seven days per week (22.3%), while others had at least one rest day. A majority of workers had many years of service. Over 70% of them had worked for more than five years in this sector and 36% had more than ten years of work experience.

### 3.2. The Prevalence of After-shift Musculoskeletal Disorder Symptoms

**Figure 1.**
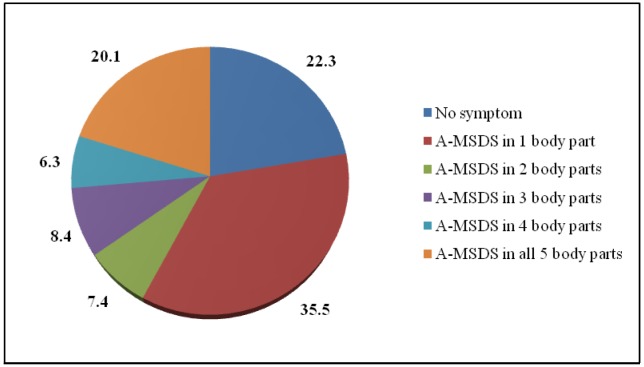
Prevalence of A-MSDS in different body parts.

Figure 1 shows that nearly four-fifths of female workers experienced A-MSDS in at least one body part (77.7%), of which 35.5% only had symptom in one random part and 20.1% experienced pain in all five parts of the body.

**Figure 2.**
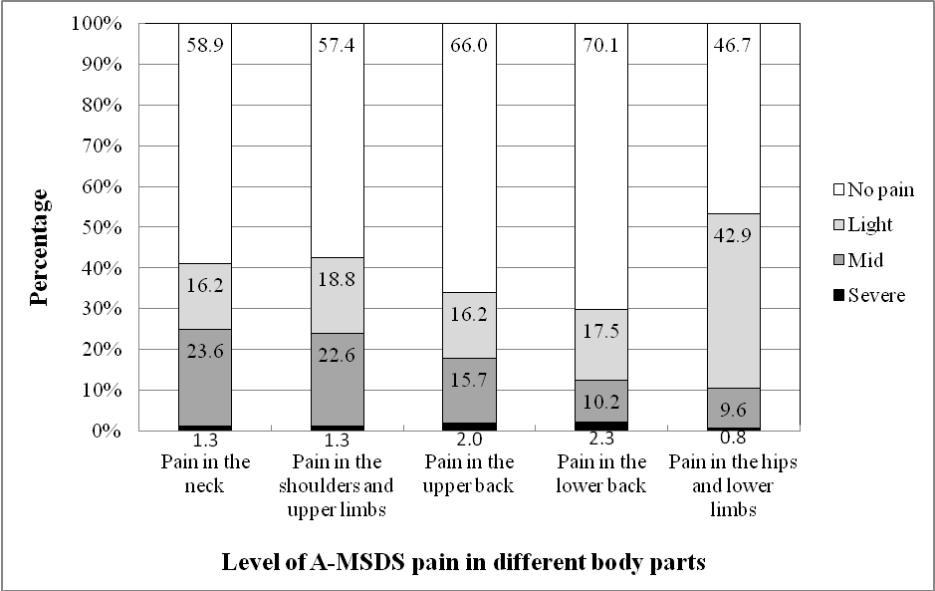
The prevalence of A-MSDS by body part and level of pain/discomfort.

The prevalence of MSDs after work in different body parts markedly varied, with the proportion of pain in the hips and lower extremities being as high as 53.3%, followed by pain in the shoulders and upper extremities (42.6%) and the neck (41.1%) (Figure 2). However, the proportions of mild pain level were highest in the neck (23.6%) and the shoulders and upper extremities (22.6%). The percentage of mild pain level in the hips and lower limbs was the lowest (9.6%). Meanwhile, A-MSDS severe pain occurred most in the lower back and upper back (2.3% and 2% respectively).

Table 1 shows that the prevalence of A-MSDS in one specific body part and in at least one random body part varied among groups with different socio-demographic characteristics. A larger number of female workers in the age groups under 30 and 41–50 years old suffered A-MSDS pain in the upper body. In addition, women having an educational level lower/higher than high school, being single or divorced, and working seven days per week also experienced greater musculoskeletal pain after work in the same body regions. Meanwhile, the distribution of A-MSDS pain in the lower body was diametrically different from that of the upper body among different socio-demographic groups. An increase of A-MSDS in all body parts was observed in workers with different years of services, with more female workers in the longer working groups being likely to experience A-MSDS in different body parts.

### 3.3. Work-related Factors in Association with After-shift Musculoskeletal Disorder Symptoms

According to the literature review, information on several working conditions, in association with the above socio-demographic status, was included in the logistic regression to better determine the contribution of work-related factors to the development of MSDs after shift in female workers in the seafood processing factory. Although the general working conditions which were observed in field study had been described in the section 2.2.3, workers' self-evaluations of their working environment were also analyzed since the tolerance levels to environmental hazards varied among individuals. The results were presented in Table 2.

**Table 1.** General characteristics of the study population and distribution of A-MSDS by body part.

Variables	Overall (n = 394)		Percentage of A-MSDS by body part (%)					
	n	%	Neck (n = 162)	Shoulders and upper limbs (n = 168)	Upper back (n = 134)	Lower back (n = 118)	Hips and lower limbs (n = 210)	At least one body part (n = 306)
**Age group (mean 35.8, SD ± 8, range 18–63)**								
≤30	105	26.6	55.2	56.2	45.7	26.7	39.0	78.1
31–40	186	47.2	29.0	29.6	24.2	23.1	64.0	80.6
41–50	85	21.6	57.6	58.8	43.5	54.1	57.6	78.8
≥51	18	4.6	5.6	22.2	22.2	5.6	5.6	38.9
**Education level**								
Secondary school and lower	108	27.4	58.3	62.0	49.1	46.3	38.9	77.8
High school	252	64	31.3	32.5	26.2	22.6	61.9	79.8
Vocational/college training	34	8.6	55.6	52.8	41.7	30.6	33.3	58.3
**Marital status**								
Single	48	12.2	62.5	62.5	43.8	31.3	27.1	70.8
Married and live with spouse	330	83.8	37.9	39.4	33.0	28.5	58.2	79.4
Widow/divorce	16	4	43.8	50.0	25.0	56.3	31.3	62.5
**Number of children**								
Not yet	62	15.7	72.9	75.0	56.3	39.6	41.7	91.7
1 or 2	308	78.2	35.5	36.7	29.4	27.9	56.1	74.8
>2	24	6.1	62.5	68.8	62.5	43.8	31.3	93.8
**Number of working days per week**								
≤6	306	77.7	32.4	34.3	25.5	20.3	49.0	76.1
7	88	22.3	71.6	71.6	63.6	63.6	34.1	83.0
**Years of working**								
≤5 years	111	28.2	45.0	51.4	37.8	26.1	28.8	62.2
6–10 years	141	35.8	19.9	22.7	19.1	17.7	62.4	78.7
11–15 years	82	20.8	45.1	40.2	39.0	31.7	67.1	85.4
>15 years	60	15.2	78.3	76.7	55.0	63.3	58.3	93.3

**Table 2.** Association of work-related factors and A-MSDS in different body parts.

Work-related factors		Overall (394)		The neck				The shoulders and upper extremities				The upper back				The lower back				The hips and lower extremities				At least one body part			
				% pain	OR		95%CI	% pain	OR		95%CI	% pain	OR		95%CI	% pain	OR		95%CI	% pain	OR		95%CI	% pain	OR		95%CI
Cold environment	Yes	339	86.0	45.1	***4.96***	***1.1***	***21.4***	45.4	1.84	0.4	7.7	36.0	1.04	0.3	3.8	32.4	1.94	0.5	7	58.7	1.11	0.3	4	82.9	0.89	0.4	2.3
	No	55	14.0	16.4	reference			25.5	reference			21.8	reference			14.5	reference			20	reference			45.5	reference		
Humid environment	Yes	292	74.1	44.2	***5.26***	***1.7***	***15.9***	44.9	***3.27***	***1.1***	***9.8***	36.3	2.30	0.9	6.1	32.2	***4.07***	***1.6***	***10.6***	63.4	0.52	0.2	1.3	85.6	0.80	0.4	1.9
	No	102	25.9	32.4	reference			36.3	reference			27.5	reference			23.5	reference			24.5	reference			54.9	reference		
Vibration	Yes	98	24.9	87.8	***10.96***	***3.5***	***33.7***	89.8	***34.41***	***9.7***	***122.6***	77.6	***31.27***	***10.4***	***94***	63.3	***6.64***	***2.8***	***15.9***	60.2	0.80	0.3	2	94.9	0.99	0.3	3.6
	No	296	75.1	25.7	reference			27.0	reference			19.6	reference			18.9	reference			51	reference			72	reference		
Harmful chemicals	Yes	147	37.3	71.4	1.25	0.5	3	70.7	1.15	0.5	2.8	58.5	1.43	0.6	3.3	50.3	0.89	0.4	2	59.2	***0.37***	***0.2***	***0.8***	95.2	***4.81***	***1.7***	***13.6***
	No	247	62.7	23.1	reference			25.9	reference			19.4	reference			17.8	reference			49.8	reference			67.2	reference		
Task requires physically strong movement	Yes	95	24.1	76.8	1.48	0.5	4.6	75.8	1.11	0.4	3.6	73.7	***2.78***	***1***	***7.5***	69.5	***4.96***	***2***	***12.6***	73.7	***3.16***	***1.2***	***8.3***	93.7	0.81	0.3	2.7
	No	299	75.9	29.8	reference			32.1	reference			21.4	reference			17.4	reference			46.8	reference			72.6	reference		
Task requires lifting heavy object or force	Yes	94	23.9	84.0	***5.49***	***1.8***	***16.7***	81.9	***4.79***	***1.5***	***15.5***	71.3	***3.56***	***1.4***	***9***	70.2	***3.68***	***1.6***	***8.3***	68.1	0.74	0.3	1.8	93.6	1.59	0.5	5.1
	No	300	76.1	27.7	reference			30.3	reference			22.3	reference			17.3	reference			48.7	reference			72.7	reference		
Awkward posture	Yes	77	19.5	83.1	2.83	0.8	10.5	80.5	1.01	0.3	3.4	54.5	0.79	0.3	2	46.8	0.68	0.3	1.6	42.9	0.64	0.3	1.5	89.6	0.75	0.3	2.3
	No	317	80.5	30.9	reference			33.4	reference			29.0	reference			25.9	reference			55.8	reference			74.8	reference		
Monotonous and repetitive task	Yes	80	20.3	81.3	***4.52***	***1.3***	***15.3***	82.5	***9.52***	***2.5***	***36.1***	48.8	0.69	88.8	1.8	47.5	***2.73***	***1.1***	***6.7***	40.0	***0.36***	***0.2***	***0.8***	88.8	1.77	0.6	5.1
	No	314	79.7	30.9	reference			32.5	reference			30.3	reference			25.5	reference			56.7	reference			74.8	Reference		
Task requires finger/wrist movements	Yes	285	72.3	50.9	2.45	0.7	8.2	51.2	1.97	0.6	6.4	38.9	0.72	91.6	2.2	36.5	0.78	0.3	2.3	66.3	0.72	0.2	2.2	91.6	***3.86***	***1.8***	***8.5***
	No	109	27.7	15.6	reference			20.2	reference			21.1	reference			12.8	reference			19.3	reference			41.3	Reference		
Shift work	Yes	285	72.3	48.4	1.58	0.6	4.4	47.7	0.74	0.3	2	38.2	1.70	87.0	4.5	33.3	0.46	0.2	1.1	64.9	***2.99***	***1.2***	***7.3***	87.0	0.81	0.4	1.9
	No	109	27.7	22.0	reference			29.4	reference			22.9	reference			21.1	reference			22.9	reference			53.2	reference		
Work more than 8 hours per day	Yes	291	73.9	46.4	0.49	0.2	1.6	47.4	1.08	0.4	3.2	34.4	0.16	87.6	0.5	34.4	1.69	0.6	4.4	64.9	***3.17***	***1.2***	***8.5***	87.6	1.48	0.7	3.3
	No	103	26.1	26.2	reference			29.1	reference			33.0	reference			17.5	reference			20.4	reference			49.5	reference		

* Marital status, number of children, years of service, and history of injury were included in the logistics regression to control bias. * Results in ***Bold and Italic***: *p* < 0.05; results in ***Bold, Italic and Underline***: *p* < 0.01.

#### 3.3.1. Cold Working Environment

Approximately 90% of the study sample reported exposure to cold at work. The prevalence of MSDs after work in female workers who were exposed to a cold environment ranged from 36% (pain in the upper back) to 58.7% (pain in the hips and lower limbs). Over four-fifths of the exposed workers experienced MSDs in at least one body part. Results showed that working in a cold environment significantly increased the risk of A-MSDS in the neck by 4.96 times (CI 95% 1.1–21.4).

#### 3.3.2. Humid Working Environment

Exposure to humidity was also reported by a large number of workers (74.1%). The proportions of A-MSDS in different body parts in workers who were exposed to a humid environment were similar to the pattern of A-MSDS in those exposed to coldness. However, humidity was significantly associated with the increased risks of A-MSDS in the neck (5.26 times), the shoulders and upper limbs (3.27 times) and the lower back (4.1 times).

#### 3.3.3. Exposure to Vibration

Only a quarter of workers reported that they were exposed to vibration at work (25%). However, the percentage of exposed workers suffering A-MSDS in different body parts were considerably high, especially pain in the neck (87.8%), the shoulders and upper limbs (89.8%) and any body part (94.9%). According to the logistic regression analysis, vibration accounted for the surging of A-MSDS risk in the neck (10.96 times), the shoulders and upper limbs (34.41 times), the upper back (31.27 times) and the lower back (6.64 times).

#### 3.3.4. Exposure to Harmful Chemicals

Among one-third of female workers claiming to be exposed to harmful chemicals at work, significant relationships were observed between this working condition and the prevalence of A-MSDS in the hips and lower limbs or in at least one body part. However, it played a role of a protection factor to the condition of MSDs in lower body parts (OR 0.37, CI95% 0.2–0.8) and increased the risk of A-MSDS in at least one part of the body (i.e. 4.81 times).

#### 3.3.5. Task Requires Physically Strong Movement or Lifting Heavy Object or Using Force

Among nearly a quarter of workers who had to perform heavy manual tasks (e.g. lifting, carrying heavy objects or/and using force), about 70% of them developed MSDs in any investigated body part and over 90% experienced MSDs in at least one body part. While general manual work increased the risk of A-MSDS in the upper back (2.78 times), the lower back (4.96 times) and the hips and lower limbs (3.16 times), object lifting particularly affected the musculoskeletal system of the upper body parts with the higher risk of MSDs in the neck (5.49 times), the shoulders and upper limbs (4.79 times), the upper back (3.56 times) and the lower back (3.68 times).

#### 3.3.6. Awkward Postures

Few workers considered themselves working in awkward postures. Statistically insignificant association was observed between posture and A-MSDS in this study.

#### 3.3.7. Monotonous and Repetitive Tasks

Although the number of workers reporting monotonous and repetitive tasks was similar to the sum of workers who reported awkward posture, results showed that this working condition significantly increased A-MSDS risks in the neck (4.52 times), the shoulders and upper limbs (9.52 times) and the lower back (2.73 times). However, it reduced the risk of A-MSDS in the hips and lower limbs (OR 0.36, CI 95%: 0.2–0.8).

#### 3.3.8. Task Requires Finger/Wrist Movements

Two-thirds of workers in the shrimp processing line reported the main use of hands and wrists at work (72.3%). This condition particularly accounted for the fourfold increased risk of experiencing A-MSDS in at least one body part (3.86 times).

#### 3.3.9. Shift Work and Number of Working Hours per Day

Similar proportions of participants informed that they worked under shift arrangement and frequently worked over 8 hours per day (72.3% and 73.9% respectively). The organization of the work schedule and duration approximately tripled the risk of A-MSDS in the hips and lower limbs in investigated female workers.

## 4. Discussion

The results showed that the characteristics of this study sample were markedly different from those in previous studies in seafood processing and light industries in China [14], Thailand [10], Myanmar [9] and even Vietnam [21], with higher proportions of female workers with older age, better educational level, married status, dependent children and longer duration of services. These personal factors might considerably contribute to the high percentage of MSDs after work in this study since various research studies had reported significant relationships between such individual characteristics and work-related MSDs in female workers [9],[10].

### 4.1. The Prevalence of After-shift Musculoskeletal Disorders among Female Workers in the Seafood Processing Company

To the knowledge of the authors, we were unable to identify any study on MSDs after shift in general and among female workers in the seafood processing industry in particular. Most studies examined work-related MSDs within 7-day or 12-month periods [9],[10],[22],[23]. However, work-related MSDs had been documented as a chronic injury which was developed through several stages after a long period of exposure to risk factors [2]. Hence, early symptoms during or after shift/working hours were a valuable indicator for timely prevention of this common health problem which imposed significant financial and health burdens on individuals and societies [1]–[6].

Findings from this study showed that a majority of female workers in the seafood processing factory were suffering from MSDs in at least one body part (77.7%). Even one-fifth of the study sample experienced A-MSDS in all investigated body parts. These numbers were higher than that of the 7-day prevalence [9],[10] but remained lower than that of the 12-month prevalence [22],[23] from previous studies on similar target groups. The high proportion of MSDs after shift in this study might be contributed by the larger proportions of female workers with older age, married status and dependent children in this study sample, such personal factors were documented to increase the risk of work-related MSDs in the above discussion.

### 4.2. Work-related Factors to the Prevalence of After-shift Musculoskeletal Disorders among Female Workers in the Seafood Processing Company

This paper analyzes the contribution of several working conditions to the development of A-MSDS with regards to potential effects from personal characteristics.

Similarly to previous studies, this study's findings elaborate the connection between cold,humid working environments and the presence of A-MSDS in the neck, shoulders and upper limbs and lower back [8],[10],[11],[22]. Various studies had reported that low temperature or high humidity had a detrimental effect on the functioning of muscles and blood vessels of the body parts in contact [11],[22]. Working in cold and humid conditions, in association with performing repetitive tasks, would increase the risk of work-related MSDs in female workers [14],[22].

Although a small number of participants were exposed to vibration or heavy manual tasks at work, these occupational hazards were responsible for the increased risk of MSDs in the upper body and back due to frequent pulling/pushing/carrying heavy objects [7],[22],[24] and awkward postures in long duration [13],[24]. In this study, participants not only possessed less musculoskeletal strength due to female weaker anatomy [22] but were also affected by older age in comparison with other studies' target groups with younger workers of both genders. These are possible explanations for the high risk of MSDs after working and its relationship with physical activities in this study.

Finally, in this study, we only found a significant relationship between work duration and A-MSDS in the hips and lower limbs. As mentioned in the section 2.2.3 on job description, female workers remained standing beside non-adjustable platforms and tables with very few observed breaks. Therefore, the musculoskeletal system in the lower half of the body was the main support for the whole body weight. Hence, the longer time spent standing would likely increase the risk of work-related MSDs [8],[25].

### 4.3. Limitations of the Study

Because of the original project's sampling procedure, which selected factories with approximately 320 workers, this paper's results are only appropriate for factories with similar conditions (i.e. large scale with more than 300 workers, modern production line, small size products such as shrimps and small fish) since the company's workforce or level of technology would determine the working conditions for the workers. Additionally, workers in seafood processing factories with larger products (such as tuna or salmon) or different items (such as crabs or seashells) could develop different work-related health problems from their distinct production lines.

Moreover, we were unable to identify validated questionnaires on MSDs after shift in Vietnamese, and the limited resources made it difficult for us to implement health examination. Therefore, we had to develop a structured questionnaire. Although the self-administrated method had been recommended as feasible and considerably effective to identify symptoms of MSDs and ergonomic exposures [26], the use of this study's newly designed instrument might produce a certain level of bias.

## 5. Conclusion

A large proportion of female workers in the seafood processing factory experienced musculoskeletal disorders after work. Physical working environment and work organization played an important role in the development of musculoskeletal disorders. The study findings imply the importance of timely improvements in the workplace to protect workers' health from risk factors of work-related MSDs.
